# Active Case Finding of Current Bornavirus Infections in Human Encephalitis Cases of Unknown Etiology, Germany, 2018–2020

**DOI:** 10.3201/eid2705.204490

**Published:** 2021-05

**Authors:** Philip Eisermann, Dennis Rubbenstroth, Daniel Cadar, Corinna Thomé-Bolduan, Petra Eggert, Alexander Schlaphof, Frank Leypoldt, Martin Stangel, Thorsten Fortwängler, Florian Hoffmann, Andreas Osterman, Sabine Zange, Hans-Helmut Niller, Klemens Angstwurm, Kirsten Pörtner, Christina Frank, Hendrik Wilking, Martin Beer, Jonas Schmidt-Chanasit, Dennis Tappe

**Affiliations:** Bernhard-Nocht-Institut für Tropenmedizin, Hamburg, Germany (P. Eisermann, D. Cadar, C. Thomé-Bolduan, P. Eggert, A. Schlaphof, J. Schmidt-Chanasit, D. Tappe);; Federal Research Institute for Animal Health, Greifswald-Insel Riems, Germany (D. Rubbenstroth, M. Beer);; University Medical Center Schleswig-Holstein, Kiel, Germany (F. Leypoldt);; Medizinische Hochschule Hannover, Hannover, Germany (M. Stangel);; Donau-Isar-Klinikum Deggendorf, Deggendorf, Germany (T. Fortwängler);; Ludwig-Maximilians-University, Munich, Germany (F. Hoffmann);; Max von Pettenkofer Institute, Munich (A. Osterman);; German Center for Infection Research, Munich (A. Osterman);; Bundeswehr Institute of Microbiology, Munich (S. Zange);; Regensburg University Hospital, Regensburg, Germany (H.-H. Niller, K. Angstwurm);; Postgraduate Training for Applied Epidemiology, Berlin, Germany (K. Pörtner);; European Centre for Disease Prevention and Control, Stockholm, Sweden (K. Pörtner);; Robert Koch Institute, Berlin (K. Pörtner, C. Frank, H. Wilking)

**Keywords:** bornavirus disease virus 1, BoDV-1, variegated squirrel bornavirus 1, VSBV-1, bornavirus, viruses, infections, serologic analysis, active case finding, case definition, etiology, epidemiology, human encephalitis, meningitis/encephalitis, zoonoses, Germany

## Abstract

Human bornavirus encephalitis is a severe and often fatal infection caused by variegated squirrel bornavirus 1 (VSBV-1) and Borna disease virus 1 (BoDV-1). We conducted a prospective study of bornavirus etiology of encephalitis cases in Germany during 2018–2020 by using a serologic testing scheme applied along proposed graded case definitions for VSBV-1, BoDV-1, and unspecified bornavirus encephalitis. Of 103 encephalitis cases of unknown etiology, 4 bornavirus infections were detected serologically. One chronic case was caused by VSBV-1 after occupational-related contact of a person with exotic squirrels, and 3 acute cases were caused by BoDV-1 in virus-endemic areas. All 4 case-patients died. Bornavirus etiology could be confirmed by molecular methods. Serologic testing for these cases was virus specific, discriminatory, and a practical diagnostic option for living patients if no brain tissue samples are available. This testing should be guided by clinical and epidemiologic suspicions, such as residence in virus-endemic areas and animal exposure.

Human bornavirus encephalitis is a severe and often fatal disease caused by 2 related zoonotic members of the family Bornaviridae, variegated squirrel bornavirus 1 (VSBV-1, species *Mammalian 2 orthobornavirus*) and Borna disease virus 1 (BoDV-1, species *Mammalian 1 orthobornavirus*). In 2015, VSBV-1 was detected as causative agent of fatal human encephalitis in a cluster of private breeders of exotic squirrels in Germany (*1*). In 2018, BoDV-1 was shown to be responsible for a cluster of transplant-related encephalitis cases (*2*) and individual encephalitis (*3*) in Germany. VSBV-1 has been detected in several holdings in Europe (private husbandries and zoologic gardens) of exotic squirrel species from the family Sciuridae of non-European descent (*4*–*6*). The geographic origin of the virus and potential additional wild animal reservoirs are unknown. In contrast, BoDV-1 is harbored by bicolored white-toothed shrews (*Crocidura leucodon*) native to Europe and is known to cause animal Borna disease (BD) after spillover infection in domestic animals in Europe. BD is a meningo-myeloencephalitis found predominantly in horses and sheep and is endemic to parts of Germany, as well as Austria, Liechtenstein, and Switzerland (*7*,*8*).

Although clinical disease and the underlying (immuno)pathology (*9*,*10*) have been described for human VSBV-1 encephalitis (*1*,*11*) and BoDV-1 encephalitis (*3*,*12*,*13*), many questions regarding the epidemiology of human VSBV-1 (*6*) and BoDV-1 (*12*) infections are still unanswered. Therefore, in March 2020, the direct detection of bornavirus infections became notifiable in Germany for humans (German Infection Protection Act [Infektionsschutzgesetz, IfSG]) and mammals (Verordnung über meldepflichtige Tierkrankheiten, TKrMeldpflV). Moreover, diagnostic external quality assurance tests are being prepared for serologic and molecular testing to equip participating laboratories with the skills and techniques to diagnose such infections and to provide reference materials in the future.

We report the results of a prospective screening study for bornavirus infections in human cases of encephalitis of unknown etiology in Germany during 2018–2020. Screening was based on a newly developed serologic testing scheme and using graded case definitions for VSBV-1 encephalitis, BoDV-1 encephalitis, and unspecified bornavirus encephalitis (i.e., bornavirus encephalitis in which the exact bornavirus species could not be determined).

## Patients, Materials, and Methods

### Patient Groups

We tested 2 patient groups. For the first group, neurologic hospital departments in Germany and researchers specializing in autoimmune encephalitis within the German Network for Research on Autoimmune Encephalitis (GENERATE, https://www.generate-net.de) were alerted about bornavirus encephalitis cases by email. In response, serum and cerebrospinal fluid (CSF) samples of patients with encephalitis of unknown etiology were sent to the Bernhard Nocht Institute for Tropical Medicine (Hamburg, Germany) during January 2018–August 2020 for analysis of possible bornavirus infections. Samples were analyzed for antibodies against VSBV-1 and BoDV-1. For the second group, serum samples from patients without a clinical history of encephalitis but for whom a bornavirus serologic analysis was nonetheless requested by the treating physicians during the same period were also analyzed.

### Serologic Testing Scheme and Case Definition

**Figure 1:**
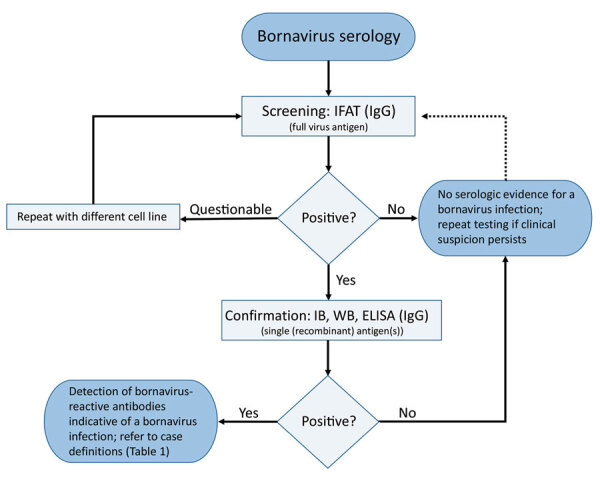
Serologic testing scheme for human bornavirus encephalitis, Germany, 2018–2020. Scheme was based on serologic screening and confirmatory assays and in conjunction with a case definition for variegated squirrel bornavirus 1 (VSBV-1) and Borna disease virus 1 (BoDV-1) encephalitis (Table 1) was diagnosed. Screening of serum samples and cerebrospinal fluid for bornavirus-reactive IgG was conducted by using an indirect immunofluorescence antibody test. A persistently BoDV-1–infected cell line was used with uninfected cells of the same cell line as controls (Vero cells or Crandell-Rees feline kidney cells). For confirmation of a positive IFAT screening result, a line blot with recombinant VSBV-1 and BoDV-1 phosphoprotein proteins was used in our study, but alternative assays, such as WB or ELISA with recombinant antigen(s) or antigen(s) derived from infected cells, might also be appropriate after sufficient validation. Adequate control serum samples from confirmed human VSBV-1 and BoDV-1 encephalitis cases and a pooled serum of 20 healthy blood donors were used for the IFAT and the line blot. IFAT, indirect immunofluorescence antibody test; IB, immunoblot; WB, Western blotting.

We developed and used a serologic testing scheme (Figure 1) in conjunction with graded case definitions (confirmed, probable, and possible cases) for human VSBV-1 encephalitis, BoDV-1 encephalitis, and unspecified bornavirus encephalitis (Table 1). We defined encephalitis or encephalopathy according to Venkatesan et al. (*14*). We performed screening of serum and CSF samples for bornavirus-reactive IgG by using an indirect immunofluorescence antibody test (IFAT) and Crandell-Rees Feline Kidney (CRFK) cells persistently infected with BoDV-1 strain V and uninfected cells of the same cell line as controls (*11*,*15*).

**Table 1:** Case definitions for BoDV-1 or VSBV-1 encephalitis/encephalopathy, Germany, 2018–2020*

Case definition
Confirmed case of BoDV-1 or VSBV encephalitis/encephalopathy
Encephalitis or encephalopathy
** AND** detection of BoDV-1 or VSBV-1 RNA in CSF or CNS tissue
** OR** detection of BoDV-1 or VSBV-1 antigen by IHC with virus-specific monoclonal antibodies in CNS tissue
Confirmed case of unspecified bornavirus encephalitis/ encephalopathy
Encephalitis or encephalopathy
** AND** detection of bornavirus antigen by IHC with cross- reactive monoclonal antibodies or polyclonal serum in CNS tissue
Probable case of BoDV-1 or VSBV-1 encephalitis/ encephalopathy
Encephalitis or encephalopathy
** AND** detection of bornavirus-reactive IgG in a serum or CSF sample by screening test (with full virus antigen, e.g., IFAT) and suitable confirmation assay detecting antibodies against individual bornavirus antigens (derived from infected cells or recombinant antigens, e.g., Western blot, immunoblot, or ELISA)
** AND **suitable comparative antibody quantification assay able to distinguish antibodies specific for BoDV-1, VSBV-1 or other orthobornaviruses (e.g., IFAT, immunoblot, ELISA)
** OR** exposure to VSBV-1-positive squirrels or epidemiologic link to BoDV-1-endemic regions
** AND **no evidence of other reasons for the clinical picture
Probable case of unspecified bornavirus encephalitis/ encephalopathy
Encephalitis or encephalopathy
** AND** detection of bornavirus-reactive IgG in a serum or CSF sample by screening test (with full virus antigen, e.g., IFAT) and suitable confirmation assay detecting antibodies against individual bornavirus antigens (derived from infected cells or recombinant antigens, e.g., Western blot, immunoblot, or ELISA)
**AND** detection of bornavirus-reactive IgG in a serum or CSF sample by screening test (with full virus antigen, e.g., IFAT) and suitable confirmation assay detecting antibodies against individual bornavirus antigens (derived from infected cells or recombinant antigens, e.g., Western blot, immunoblot, or ELISA)
Possible case of BoDV-1 or VSBV-1 encephalitis/encephalopathy
Encephalitis or encephalopathy
** AND** residence in BoDV-1-endemic area or exposure to VSBV-1–positive squirrels
** AND** no evidence of other reasons for the clinical picture

*Case definitions replace those of Tappe et al. (*6*). Encephalitis or encephalopathy was defined according to Venkatesan et al. (*14*). BoDV-1, Borna disease virus 1; CNS, central nervous system; CSF, cerebrospinal fluid; IFAT, immunofluorescence antibody test; IHC, immunohistochemical analysis; VSBV-1, variegated squirrel bornavirus 1.

Because of high antigenic cross-reactivity within the genus *Orthobornavirus*, the BoDV-1 IFAT also detects antibodies against VSBV-1 (*1*,*11*,*16*). All serum or CSF samples with intranuclear IFAT patterns indicative for bornavirus infections (*11*,*15*) at dilutions >1:10 were considered positive. End-point titers are indicated as the reciprocal value of the highest positive dilution factor. We used a line blot (immunoblot) with recombinant phosphoprotein (P) from VSBV-1 and BoDV-1 as a confirmatory assay (*11*,*15*). The P protein was chosen because it was shown to be more specific than the nucleoprotein for serologic analysis (*11*). The cutoff value of the line blot was 16 arbitrary units per antigen, as validated by the manufacturer (Euroimmun, https://www.euroimmun.com) by using bornavirus-positive serum and CSF samples in comparison to >200 controls without evidence of bornavirus encephalitis. In our study, we used serum samples from laboratory-confirmed human VSBV-1 and BoDV-1 encephalitis cases as positive controls and pooled serum samples from 20 healthy blood donors as negative control for both the IFAT and the line blot.

### Molecular Assays and Cell Culture

We performed VSBV-1–specific (*1*) and BoDV-1–specific (*2*) quantitative reverse transcription PCRs (qRT-PCRs) for CSF and brain tissue of seropositive patients, if available. In positive cases, we used next-generation sequencing (NGS) to generate full-length virus genomes (*1*,*11*,*12*). We performed virus isolation in Vero or CRFK cells (*12*) and confirmed by direct immunofluorescence test using polyclonal antibodies (*11*,*12*) against VSBV-1 and BoDV-1.

### Ethics

The planning, conduct, and reporting of this study was in accordance with the Declaration of Helsinki, as revised in 2013. Ethical clearance was obtained from the Medical Board of Hamburg (no. PV5616).

## Results

For group 1, serum and CSF samples from 103 patients with encephalitis (at that time of unknown etiology) were received and tested during the study. Samples were from 60 male and 43 female patients; age range was 1–89 years (median age 48 years). For group 2, bornavirus serologic analysis was conducted for serum samples from 121 patients who had no clinical history of encephalitis but for whom bornavirus serologic analysis was requested. Samples were from 55 male and 66 female patients; age range was 4–84 years (median age 45 years).

**Figure 2:**
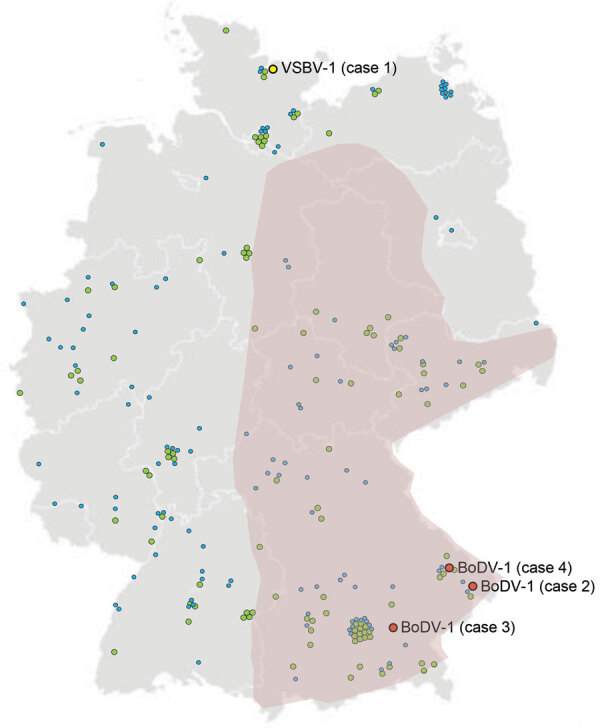
Germany showing locations of residences of human case-patients who had encephalitis and other conditions and were tested for bornavirus etiology, 2018–2020. Among 103 encephalitis cases with unknown etiology, 4 bornavirus cases were found: 1 chronic VSBV-1 infection in northern Germany (case 1) and 3 BoDV-1 infections in southern Germany (cases 2, 3, and 4). Encephalitis cases without a bornavirus etiology are indicated as green circles. Among 121 cases without a clinical history of encephalitis but for whom a bornavirus serologic analysis was requested, no bornavirus infections were detected (blue circles). Purple indicates regions known to be endemic for BoDV-1. BoDV-1, Borna disease virus 1; VSBV-1, variegated squirrel bornavirus 1.

**Figure 3:**
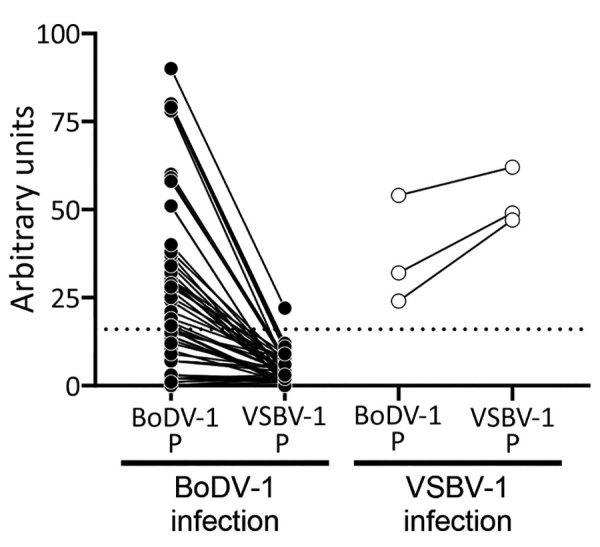
Reactivity of samples from BoDV-1- and VSBV-1-infected patients with homologous and heterologous bornavirus P antigens, Germany. Bornavirus indirect immunofluorescence antibody test–positive serum and cerebrospinal fluid samples were tested by using the Euroimmun (https://www.euroimmun.com) line blot with BoDV-1 P and VSBV-1 P antigens (BoDV-1: 52 samples from 14 patients; VSBV-1: 3 samples from 2 patients). Samples originated from patients with laboratory-confirmed BoDV-1 or VSBV-1 infection (*2*,*10**–**12*; this study). Results are indicated as arbitrary units. Dotted line indicates cutoff value of 16 as defined by the manufacturer. BoDV-1, Borna disease virus 1; P, phosphoprotein; VSBV-1, variegated squirrel bornavirus 1.

For group 1, a total of 4 (3.9%) confirmed bornavirus encephalitis case-patients were detected: 1 VSBV-1 case-patient in northern Germany (Schleswig-Holstein) (case-patient 1) and 3 BoDV-1 case-patients in Bavaria in southern Germany (case-patients 2–4; Figure 2). None of these case-patients had a travel history outside Germany in the 5 months before symptom onset. Initial testing by IFAT resulted in detection of bornavirus-reactive antibodies in serum and CSF samples for all 4 case-patients (Table 2). Samples of case-patients 1–3 also showed positive results for a bornavirus P line blot assay. Serum and CSF samples from case-patient 1 showed higher signals for VSBV-1 P than BoDV-1 P, and serum samples from case-patients 2 and 3 showed higher signals for BoDV-1 P (Table 2). The discriminatory potential of this test was confirmed by analysis of reference serum samples from several laboratory-confirmed BoDV-1 and VSBV-1 encephalitis cases (Figure 3). qRT-PCR and full-genome sequencing confirmed the bornavirus infection for all 4 case-patients.

**Table 2:** Line blot results for 4 case-patients who had bornavirus encephalitis, Germany, 2018–2020*

Case no.	Virus	Serum				Cerebrospinal fluid		
		IFAT	VSBV-1 P	BoDV-1 P		IFAT	VSBV-1 P	BoDV-1 P
1	VSBV-1	**655,360**	**62**	***54***		**20,480**	**49**	***32***
2	BoDV-1	**640**	*9*	**30**		**80**	*2*	14
3	BoDV-1	**2,560**	*4*	**17**		**160**	*2*	2
4	BoDV-1	**2,560**	*2*	1		**160**	*1*	0

*Cutoff value is 16 arbitrary units per antigen; positive results are indicated in bold. Results of antibody testing against heterologous antigens are indicated in italics. BoDV-1, Borna disease virus 1; IFAT, immunofluorescence antibody test; P, phosphoprotein; VSBV-1, variegated squirrel bornavirus 1.

### Case-Patient 1: Chronic VSBV-1 Encephalitis/Encephalopathy

Case-patient 1 was a 41-year-old man, a former zoo animal caretaker, from northern Germany who was given a diagnosis of VSBV-1 infection at the Bernhard Nocht Institute for Tropical Medicine during February 2019. Acute encephalitis had developed in the patient during in 2007; by 2019, he was living in a nursing home and showed severe neurologic deficits and disabilities. The patient had cared for exotic squirrel species in the same zoo (zoo D [*6*]) in which a woman (another zoo animal caretaker) showed development of VSBV-1 encephalitis during 2013 after contact with exotic squirrels; her case had been retrospectively detected in 2018 (*11*). 

IFAT titers for case-patient 1 in February 2019 were extremely high (655,360 for serum and 20,480 for CSF). Line blot results for antibodies against VSBV-1 P were 62 units for serum and 49 units for CSF (Table 2). qRT-PCR results were negative for VSBV-1 in stored CSF obtained during 2007 but positive in an archived formalin-fixed brain biopsy specimen obtained the same year (cycle quantitation [Cq] value 30.9). Five months after the diagnosis of VSBV-1 en-cephalitis, the patient died of urosepsis, 12 years after onset of neurologic illness.

### Case-Patient 2: Acute BoDV-1 Encephalitis

**Figure 4:**
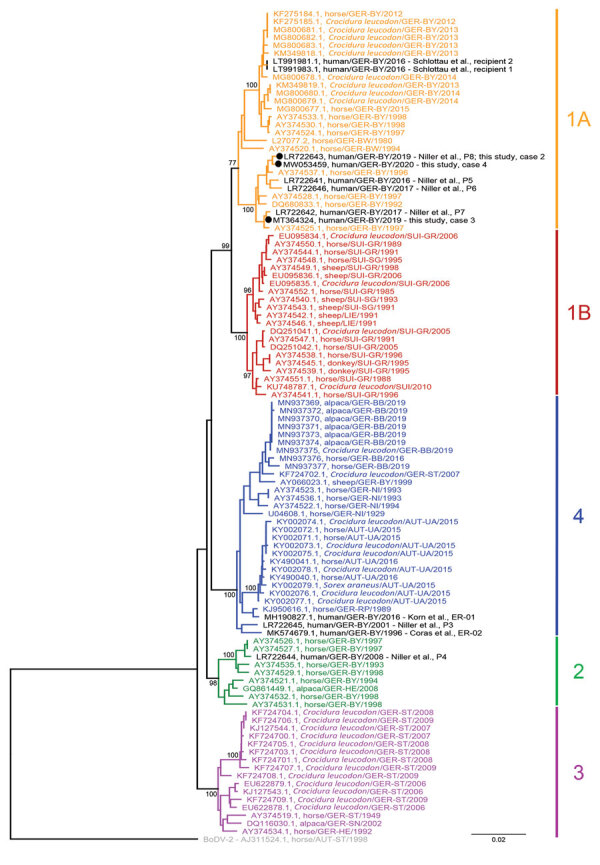
Phylogenetic analysis of BoDV-1 nucleotide sequences from virus-endemic areas, Germany. Shown are partial bornavirus sequences (nucleoprotein gene to phosphoprotein gene, 1,824 nt, representing genome positions 54–1877 of BoDV-1 reference genome U04608), including BoDV-1 sequences from animals and humans in virus-endemic regions in Germany, Austria, Switzerland, and Liechtenstein. BoDV-2 was used as an outgroup. Analysis was performed by using the neighbor-joining algorithm and the Jukes–Cantor distance model in Geneious Prime (https://www.geneious.com) and the tree was rooted for the VSBV-1 clade. Human sequences are indicated in black. Sequences of cases 1–4 included in this study are indicated in bold. Values at branches represent support in 1,000 bootstrap replicates. Only bootstrap values >70 at major branches are shown. Cluster designations, host, and geographic origin are indicated according to previously published studies (*2**,**7**,**8**,**12**,**17**–**23*). Colors and numbers at right indicate clusters. Scale bar indicates nucleotide substitutions per site. AUT, Austria: UA, Upper Austria; ST, Styria. GER, Germany: BB, Brandenburg; BW, Baden-Wuerttemberg; BY, Bavaria; HE, Hesse; NI, Lower Saxony; RP, Rhineland-Palatinate; SN, Saxony; ST Saxony-Anhalt. LIE, Liechtenstein. SUI, Switzerland: GR, Grisons; SG, St. Gall.

Case-patient 2 was a 55-year-old woman, a part-time cleaner from Bavaria who was given a diagnosis of BoDV-1 infection in February 2019. The patient had encephalitis, fever, headache, and coma develop in mid-January 2019. IFAT titer was 640 for serum and 80 for CSF. Line blot results for antibodies against BoDV-1 P were 30 units for serum and 4 units for CSF (Table 2). A qRT-PCR result for BoDV-1 in CSF was weakly positive (Cq 35.3). Case-patient 2 died 1 day after diagnosis and 3 weeks after onset of disease. Postmortem virus isolation and sequencing from brain tissue were successful (GenBank accession no. LR722643; Figure 4) (*12*). This case-patient was included in a recent case series from Bavaria as case-patient P8 by Niller et al. (*12*), and histopathologic results were described in detail as case-patient 6 by Liesche et al. (*10*).

### Case-Patient 3: Acute BoDV-1 Encephalitis

Case-patient 3 was an 11-year-old girl from a ru-ral region of Bavaria who was given a diagnosis of BoDV-1 infection in November 2019. The patient had encephalitis, fever, headache, and epileptic seizures develop during mid-October 2019. IFAT titer was 2,560 for serum and 160 for CSF. Line blot results for antibodies against BoDV-1 P were 17 units for serum and 2 units for CSF (Table 2). A qRT-PCR result for BoDV-1 in CSF was positive (Cq 33.0). A full-length virus genome was obtained by NGS from brain tissue attached to a CSF pressure probe removed after death (GenBank accession no. MT364324; Figure 4). Virus isolation from brain material was successful in CRFK cells after 2 passages of the inoculated cells. Case-patient 3 died 2 days after diagnosis and 4 weeks after onset of illness. No autopsy was performed.

### Case-Patient 4: Acute BoDV-1 Encephalitis

Case-patient 4 was a 79-year-old man, a farmer from a rural region of Bavaria who was given a diagno-sis of BoDV-1 infection in June 2020. The patient had encephalitis, fever, and confusion developat the end of May 2020. IFAT titer was 2,560 for serum and 160 for CSF. Line blot results for antibodies against BoDV-1 P were 1 unit for serum and 0 units for CSF (Table 2). A qRT-PCR result for BoDV-1 in CSF was positive (Cq 34.0). Virus isolation from CSF was not successful, but a full-length virus genome was obtained by NGS (GenBank accession no. MW053459; Figure 4). Case-patient 4 patient died 1 day after diagnosis and 4 weeks after onset of illness. No autopsy was performed.

All 4 case-patients fulfilled the case definition for confirmed VSBV-1 or BoDV-1 encephalitis. No unspecified bornavirus encephalitis cases and no probable or possible cases were identified in this study. Consistent with their geographic origin, we found that BoDV-1 sequences from case-patients 2–4 (GenBank accession nos. LR722643, MT364324, and MW053459) were closely related to human- and animal-derived sequences in BoDV-1 cluster 1A from home regions of the patients in southeastern Bavaria (Figure 4). Case-patients 2 and 4 lived <50 km from each other (Figure 2). Their BoDV-1 genome sequences showed higher nucleotide identity to each other (99.6%; Figure 4) than to the viral sequence obtained from patient 3 (98.6%), who lived ≈80 km southwest of case-patients 2 and 4. In congruence with previous confirmed human BoDV-1 infections, phylogenetic analysis suggested that zoonotic transmission occurred from the natural reservoir of the viruses near the most recent residences of the patients (*2*,*12*). For the nonencephalitis patients in group 2, we found no persons who had bornavirus-reactive antibodies in the IFAT or the IFAT plus line blot (Figure 2).

## Discussion

The epidemiology of human bornavirus encephalitis is still largely unknown. In our prospective study, we identified 4 bornavirus case-patients (3.9%) in a group of 103 case-patients who had cryptic encephalitis. The VSBV-1 case-patient had a disease course during 2007–2019, whereas the BoDV-1 case-patients died from acute infection during 2019 and 2020. Regarding incidence of infection, bornavirus encephalitis cases caused by VSBV-1 and BoDV-1 appear to be rare. Cases were only found among the group of encephalitis patients and not among patients with unspecified symptoms for whom a bornavirus serologic analysis was nonetheless requested. This finding supports previous studies that scientific evidence for a possible bornavirus etiology for other clinical entities apart from encephalitis continues to be lacking (*15*,*17*,*18*).

The cases detected in our study confirm previously identified risk factors: acquiring VSBV-1 encephalitis through contact with exotic squirrel species and association of BoDV-1 encephalitis with residence in mainly rural environments in areas endemic for animal BD and thus BoDV-1. The incidence of human VSBV-1 continues to be restricted to the small group of zoo animal caretakers and squirrel owners exposed to infected exotic squirrels. The denominator of this group is unknown, but small. Known confirmed human case-patients include 3 private breeders (*1*) and 2 zoo animal caretakers (case-patient 1 reported here; case reported in *11*). All of these patients died, although the patient identified in this study survived for 12 years after the acute phase. In another study (*6*), a squirrel breeder had a serologically positive result after recovery from a transient neurologic disease. This case was classified as a probable case because diagnostic material suitable for confirmation of the infection by direct virus detection (Table 1) was not available. Two additional breeders identified in the same study had died of unclear encephalitis, but no archived diagnostic materials were available. These cases were categorized as possible cases (Table 1).

The incidence of diagnosed BoDV-1 encephalitis cases is currently ≈2 cases/year in Germany; there is a strong restriction to known areas to which BoDV-1 is endemic (*12*). In these regions, BoDV-1 might be a major cause of previously cryptic encephalitis: In a recent study of archived brain tissue material from patients who had fatal encephalitis without a known cause from 1 center in Bavaria in an area to which animal BD is endemic, 7 (78%) of 9 had a BoDV-1 infection (*12*). At the same time, a seroprevalence study performed in BoDV-1–endemic areas found only 1 seropositive person among a presumed risk group of 736 veterinarians (0.14%; clinical and diagnostic follow-up was not possible) and none among 373 healthy blood donors (0%; *15*). Thus, these data suggest that BoDV-1 infection and BoDV-1–induced encephalitis are rare, even in virus-endemic areas, but have a high case-fatality rate.

Bornavirus encephalitis cases caused by VSBV-1 and BoDV-1 are clinically similar, severe, and in nearly all reported cases fatal. The course of BoDV-1 encephalitis appears to be more rapid (*1*–*3*,*10*–*13*). All but 1 case-patient who had confirmed bornavirus encephalitis died; only in a transplant-associated BoDV-1 encephalitis cluster did 1 patient survive but had neurologic sequelae (*2*). In our study, all 4 patients died of the disease; however, a patient who had severe VSBV-1 encephalitis/encephalopathy had a 12-year chronic course of disease. More thus far undetected chronic bornavirus infections in persons who have neurologic deficits after encephalitis or chronic encephalopathy might be present and should undergo diagnostic testing for a bornavirus etiology. Clinical awareness, particularly for severe encephalitis cases in areas to which BoDV-1 is endemic or after contact with exotic squirrel species, should lead to early testing for a possible bornavirus etiology while the person is still alive. Bornavirus infection needs to become a routine target for differential diagnostics. An early diagnosis would be a prerequisite for antiviral chemotherapy.

Following graded case definitions for VSBV-1, BoDV-1, and unspecified bornavirus encephalitis/encephalopathy (Table 1), we used a serologic testing scheme (Figure 1) for rapid initial intra vitam diagnosis of bornavirus encephalitis. Positive serologic results were subsequently confirmed by direct pathogen detection for all 4 bornavirus encephalitis cases. In this study, as well as in previous studies, higher and earlier detectable antibody titers were usually observed in serum samples than in CSF samples (*2*,*12*,*13*). The time of seroconversion during human bornavirus encephalitis is variable; some patients are already seropositive at the time of hospitalization, and others show development of detectable antibodies only shortly before death (*2*,*12*). Thus, serologic follow-up testing (likely within days) should be performed in encephalitis case-patients who have epidemiologic risk factors but initially negative results because such patients might be infected with zoonotic bornaviruses but might not yet have seroconverted.

IFAT appears to be more sensitive than the line blot assay used in this study and previous studies (*12*). In this study, line blot results were below the cutoff value for case-patient 4 in serum and CSF samples. This finding emphasizes the need for serologic follow-up testing for some case-patients, with the expectation that the screening test result will be confirmed by positive single-antigen assay results in follow-up samples that have increased antibody titers. Without the molecular confirmation, case-patient 4 would have been classified as having a possible case, stressing the need for molecular testing. In addition to sensitivity, specificity is a crucial issue of serologic testing for bornavirus infections, emphasizing the need for careful evaluation of IFAT results with a specific granular intranuclear pattern observed only in the bornavirus-infected cells (*2*,*11*,*15*,*16*).

Orthobornaviruses show considerable cross-reactivity among each other (*16*). Furthermore, antibodies against individual antigenic epitopes might be detected despite lack of any known previous bornavirus contact, leading to false-positive results (*19*). Establishing and optimizing assays such as Western blot, line blot, or ELISA, for detection of antibodies against individual bornavirus antigens are needed to enable subsequent confirmation of positive IFAT results and help to partly overcome the shortcomings of bornavirus serologic analysis. Furthermore, comparative testing by using antigens from different orthobornaviruses by IFAT (*16*) or line blot (as shown in this study) can enable discriminatory prediction of VSBV-1, BoDV-1, or other orthobornavirus infections. In the line blot used in this study, serum samples from VSBV-1–infected and BoDV-1–infected patients reliably showed higher arbitrary antibody units for the respective homologous P antigen than for heterologous antigen (Table 2). This provisional discrimination might be useful for prognosis because BoDV-1 infections appear to be more rapidly fatal than VSBV-1 infections. Despite these measures, incidental, singular, bornavirus-reactive antibodies might remain indistinguishable from truly positive results.

Because of these limitations of serologic testing, direct pathogen detection is mandatory to confirm of the initial serologic diagnosis and discrimination of VSBV-1 and BoDV-1 infections, as indicated in the graded case definitions (Table 1). However, intra vitam direct detection of the virus is severely hampered by the strong cell-associated and neurotropic nature of the virus and its almost exclusive restriction to the central nervous system in dead-end hosts, such as humans (*2*,*11*,*12*). The virus is not detectable in blood, and viral RNA detection by qRT-PCR in CSF samples often shows negative or only weakly positive results; the negative predictive value appears to be low (*12*). Bornavirus RNA detection in biopsy specimens from affected brain areas seems to be the most sensitive diagnostic method, but the difficulties with these procedures are high and unlikely to be met early in the disease course. Postmortem, unequivocal confirmation of infection can be made by using qRT-PCR, in situ hybridization, and immunohistochemical analysis of brain tissue (*2*,*3*,*9*–*13*). Sequencing the viral genome and subsequent phylogeographic analysis might provide information on the regional source of infection (*2*,*12*).

The first limitation of this study is that, although it was a large screening program of encephalitis cases for bornavirus infections, a complete or representative sampling was not performed. Thus, the prevalence of human bornavirus encephalitis remains to be investigated. Second, only a few bornavirus cases were found, which was partly caused by the overall low incidence of bornavirus encephalitis in humans. This limitation also emphasizes the need for increased awareness for this disease. Detection of future cases will provide further opportunity to approve the herein proposed case definition criteria. Third, only encephalitis cases from Germany were investigated in this study. However, the virus-endemic region for BoDV-1 also includes neighboring Austria, Liechtenstein, and Switzerland. Undetected human BoDV-1 cases are also expected in these countries.

In conclusion, human bornavirus encephalitis cases remain rare in the general population in Germany, even for BoDV-1 infections in areas to which animal BD is endemic. Human bornavirus encephalitis is often fatal. Chronic cases occur at least for VSBV-1 infections, but might be exceptional. There is no evidence that zoonotic bornaviruses might cause diseases other than encephalitis (e.g., encephalomyelitis, encephalomyeloradiculits) in humans. Antibody testing in serum samples is sensitive but requires confirmation by direct detection of virus. The proposed testing scheme and case definitions proved useful. All patients who have encephalitis (especially a severe course) from virus-endemic areas or after contact with exotic squirrels should be tested for a bornavirus infection, ideally early in the disease course by antibody testing in serum samples (*21*–*23*).
